# Quantitative assessment of a data-limited recreational bonefish fishery using a time-series of fishing guides reports

**DOI:** 10.1371/journal.pone.0184776

**Published:** 2017-09-11

**Authors:** Rolando O. Santos, Jennifer S. Rehage, Aaron J. Adams, Brooke D. Black, Jason Osborne, Emily K. N. Kroloff

**Affiliations:** 1 Earth & Environment Department, Florida International University, Miami, Florida, United States of America; 2 Southeast Environmental Research Center, Florida International University, Miami, Florida, United States of America; 3 Bonefish and Tarpon Trust, Coral Gables, Florida, United States of America; 4 Everglades National Park, USNPS/SFNRC, Homestead, Florida, United States of America; Department of Agriculture and Water Resources, AUSTRALIA

## Abstract

Recreational fisheries can be prone to severe declines, yet these fisheries, particularly catch-and-release, are often data-limited, constraining our ability to conduct stock assessments. A combination of catch and effort indices derived from fisheries-dependent data (FDD) gathered from fishing logbooks could be a powerful approach to inform these data gaps. This study demonstrates the utility of using different catch metrics such as indices of abundance, species richness associated with reported catch, and the success rate of targeted trips, to assess historical shifts in the trajectory of the data-limited bonefish (*Albula vulpes*) fishery in Florida Bay, an economically-important recreational fishery within the Caribbean Basin. We used FDD from fishing guide reports submitted to Everglades National Park to determine temporal patterns in the bonefish population over the past 35 years. These reports indicated a decline in recreational catches in Florida Bay since the late 1980s, with an accelerated decline starting in the late 1990s-early 2000s. Analyses showed an overall 42% reduction in bonefish catches. Trends in the proportion of positive trips (i.e., the probability of catching success) followed the declining catch patterns, suggesting major population changes starting in 1999–2000. As bonefish catches declined, species richness in bonefish trips increased by 34%, suggesting a decrease in bonefish abundance and/or shift in fishing effort (e.g., giving-up time, changes in preferred species). Results provide additional resolution to a pattern of decline for bonefish in South Florida and highlight the value of reconstructing time-series for the development of hypotheses about the potential driving mechanisms of species decline. Further, the data-limited nature of most recreational fisheries, and the increase in a use of catch-and-release as a fisheries management strategy point to the need to develop further data integration tools to assess population trends and the sustainability of these fishery resources.

## Introduction

Over the last decade, there has been a recognition that recreational fisheries, including catch-and-release fisheries, can be subject to population collapse and stock depletion [1–4]. Recreational fisheries resilience has been compromised by numerous factors, including increasing pressure from competing commercial and artisanal fisheries, and threats such as intensified coastal land use, habitat and hydrological modification, pollution, eutrophication, hypoxia, and species invasions [5]. For example, in northern Australia, species-specific studies have shown the importance of the quantity and timing of freshwater inflows to coastal fisheries production [6]. Recreational salmon fisheries in the USA have also been affected by the modification of river networks and watershed land-use changes [5]. In Florida (USA), a combination of extreme weather events and intense recreational pressure have induced major declines in common snook (*Centropomus undecimalis*) stocks, leading to the implementation of management measures that ensure the viability of this fishery [7,8]. At the same time, seagrass and coral reef habitat loss have been linked to the decline of recreational fish populations throughout the Atlantic coast of the USA and the Caribbean [9–11]. Finally, recreational harvest and catch-and-release practices have been identified as a prime source of population declines for some species (e.g., trout, walleye, red drum) [1,4,12], or have been found to interact with habitat disturbances and deterioration effects already operating to negatively affect targeted species [4,13].

Extensive quantitative data are needed to assess recreational fisheries stocks, reconstruct historical abundance trends, and determine factors regulating their population levels, yet frequently, data to conduct effective stock and harvest assessments are lacking [14]. Fisheries-dependent data (FDD) from mandatory catch return cards, logbooks, sale slips or interviews often represent the only available data source, providing estimates of abundance needed for temporal trend assessments [5,15,16]. FDD can be analyzed with statistical models to generate estimates of catch, effort, and catch-per-unit-effort (CPUE) that inform individual and multispecies stock assessments [15,17,18]. In addition, FDD have been successfully used to assess how disturbance events (e.g., extreme climate events, fishing-related disturbances) influence ecological processes, community resilience dynamics, and regime shifts in fisheries [19–22]. FDD may be considered a traditional data source in fishery science, and often can be a powerful tool to inform data-limited fisheries, particularly when long time-series are available [15]. In this study, fishing guides catch reports were used to reconstruct temporal dynamics for the data-limited bonefish (*Albula vulpes*) recreational fishery in Florida Bay, and to make inferences about changes in bonefish abundance, particularly in relation to potential drivers of decline.

Bonefish constitute an economically-important fishery throughout the Caribbean [23–27]. In South Florida, where the fishery is exclusively catch-and-release and bonefish are a key part of a popular flats fishery that focuses on sight fishing in shallow seagrass habitats, it is estimated that one bonefish is worth $3,500, with a possible lifetime worth of approximately $75,000 (i.e., based on a maximum age of 20 years)[26]. A recent economic assessment estimated that approximately $466 million of the total economic impact of saltwater angling in Florida is generated by the Florida Keys flats fishery alone [25]. Yet, despite this high value both locally and regionally, the availability of stock assessments and bioecological studies are limited, and key data on spawning and recruitment dynamics, habitat use patterns, and life history remain unknown [23,27] (i.e., data-limited fishery).

We propose that this data-limited recreational fishery would benefit from studies that comprehensively assess its resilience, particularly given that numerous stressors may increasingly jeopardize the sustainability of the fishery. For example, fishing effort targeting bonefish throughout Florida Keys and Florida Bay has been increasing over the last several decades [2,23]. At the same time, coastal environments in Florida Bay, some of which constitute essential habitat for bonefish (i.e., foraging grounds, nursery habitats, spawning aggregation areas), have been subject to a series of anthropogenic disturbances [28], largely associated with altered freshwater deliveries throughout the Everglades watershed [29]. These disturbances, in combination with natural droughts, have caused hypersalinity and seagrass die-off events that have impacted up to 30% of Florida Bay (i.e., 1987–89 and 2015 seagrass die-offs [28,30]). These events have caused marked state shifts in Florida Bay, unleashing a cascade of ecological effects including epibenthic community loss and shifts in structure, algal blooms, sponge mortality, and reductions in shrimp and spiny lobster landings [28,31,32]. However, our understanding of the effects of these major events and other extreme climate events on economically-valuable recreational fisheries such as bonefish remains unknown [22]. The socio-economic importance of the Florida Bay bonefish stock and the high demand it experiences by recreational anglers, highlight the need to assess temporal trends in catch (i.e., gradual vs. breakpoint changes) and identify possible drivers of population state, decline or recovery.

Recently, fishing guides in Florida Bay and the Florida Keys have reported a concerning decline in bonefish numbers [23,33], thus adding to the list of recreational fisheries in decline due to a myriad of anthropogenic and environmental factors [8,12,27,34]. These assessments of a decline have been largely based on qualitative data that stem from anglers’ perceptions and experiences (i.e., local ecological knowledge, [2,33,35, but see 23]). Thus in our study, we built a retrospective bonefish catch timeline over the past 34 years using FDD from fishing guide reports with the objective of quantitatively assessing temporal trends of bonefish fishery patterns (i.e., annual catch, catching success, and catch species richness) in Florida Bay and to identify major shifts in temporal patterns. Given the present and past environmental events affecting Florida Bay (e.g., seagrass die-offs, algae blooms) and the reported increase in fishing pressure in South Florida and the Caribbean region, we hypothesized that the bonefish catch trend would display drastic shifts and nonlinear declining patterns that likely reflect disturbance events that degraded the spatial cover and quality of bonefish habitats [4].

## Materials and methods

### Study domain

**Fig 1:**
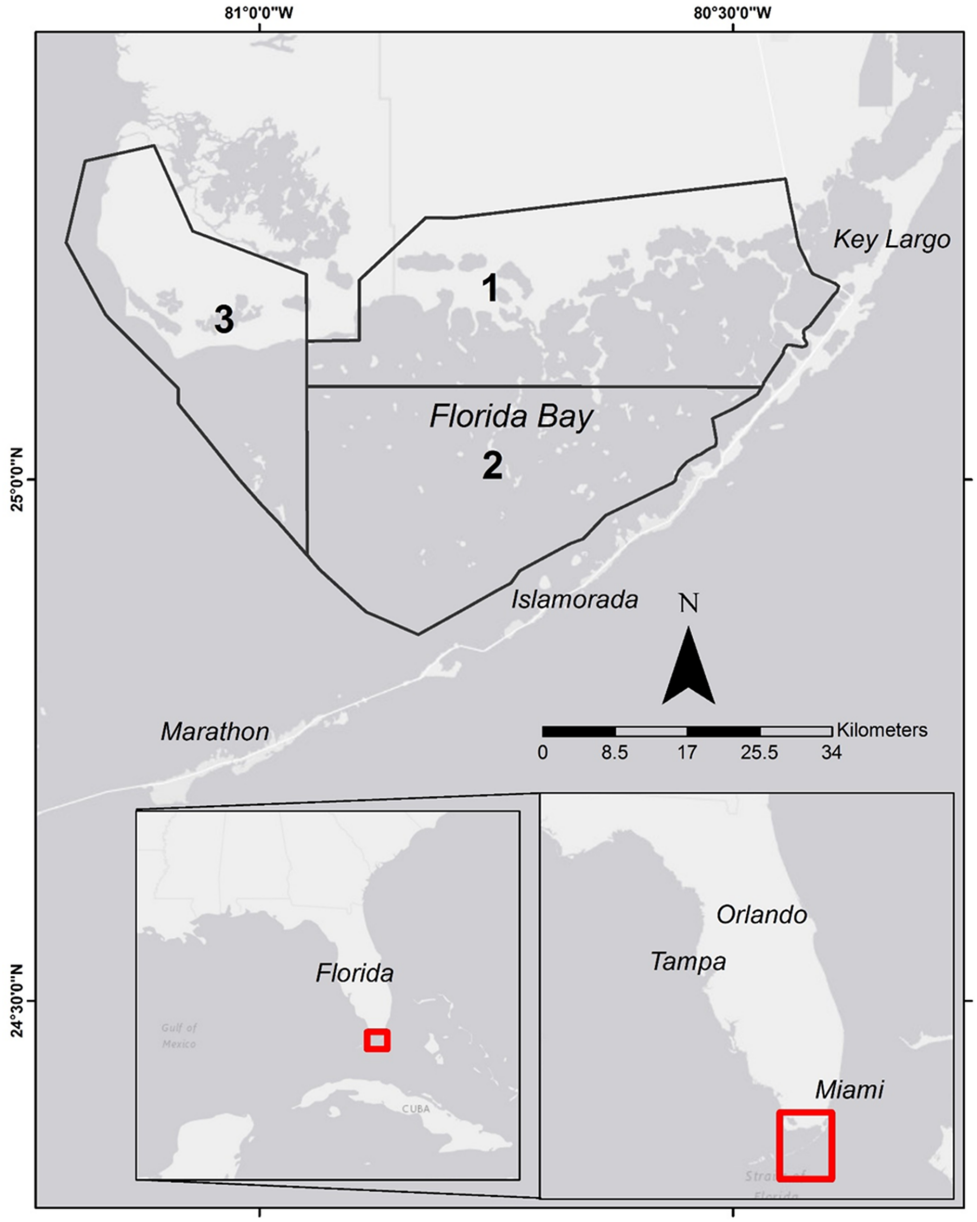
Map of Florida Bay in the Southern Everglades National Park (black dotted line) and Florida (USA). Polygons indicate the 3 guide reporting areas (1–3) used in analyses.

We examined temporal trends in the bonefish flats recreational fishery in Florida Bay, a shallow, subtropical estuarine lagoon located in the southern end of the Greater Everglades drainage and Everglades National Park (ENP, Fig 1). The focal study area also included the ‘bay side’ of the upper Florida Keys, from Key Largo to Long Key. Recreational fishing is a key economic activity in the region, with one in five Florida anglers fishing the Everglades region, generating $1.5 billion in economic activity, and with bonefish being one of the top targeted species [24]. Our focus was in Florida Bay since this is the area where the documented bonefish decline is the greatest, and is historically a major fishing ground for the species [2,23,33]. The exact mechanisms driving the decline in Florida Bay are unknown, yet concerning, given the key role of bonefish as an overall indicator of ecosystem health [23,36], the large socio-economic value of this recreational fishery to the Florida Keys [25,26], and environmental events affecting the region (e.g., seagrass die-off and algal blooms, [28,30,31]). Bonefish diet, life histories, and habitat use are closely linked to seagrasses resources [37,38], which are of vital importance to coastal ecosystem functioning [39–41].

### Data collection

To elucidate and assess temporal trends in bonefish catches, we used FDD obtained from professional guide logbooks. These types of data sets have pros and cons, as well as inherent biases [15]. However, FDD are often the only available information to assess long-term changes in species abundance and distribution, especially in data-limited fisheries such as recreational and catch-and-release fisheries [15,16,18,42]. The FDD used in this study were derived from professional fishing guides operating within ENP. Guides are required to obtain an annual permit from ENP and report their monthly catch and effort on a per trip basis via logbooks. Specifically, guides reported the number of fish kept and released per species, effort (number of anglers, hours fished), and main species targeted (i.e., the primary species that was targeted in the trip) [43] within 6 fishing areas (see [44,45] for additional details on methodology). For this study, we used the data reported in fishing areas within Florida Bay’s geographical boundaries (Fishing Area 1–3, Fig 1) because bonefish occur only in this area of ENP. The FDD used in our analyses were obtained from the National Park Service via their Marine and Estuarine Resource Management Program (https://www.nps.gov/ever/learn/nature/marine.htm). We used guide reports for the period 1980 to 2014 (n = 34 years), totaling 5,039 guide reports that reported on bonefish and averaging 144 bonefish reports per year. For all analyses, catch and effort data were aggregated to monthly totals to smooth daily variation due to weather, differences in fishing activity between weekends and weekdays, and other temporal factors.

#### Statistical analyses

Prior to any inference about abundance trends, the catch data were subject to a standardization procedure in order to account for potential biases, such as spatial and temporal dynamics in effort allocation, fishing behavior and tactics, and species composition [15,16]. Our standardization procedure primarily relied on Generalized Additive Models (GAMs) to standardize the catch and effort data reported by guides. GAMs have been used in various studies to standardize CPUE [16,46,47], and are especially useful for incorporating multi-tactic spatial effects (e.g., changing fishing gear preference across space; [16]), exploring non-linear relationships [48] and identifying ecological thresholds [49].

A GAM is a semi-parametric extension of the generalized linear model that includes a linear predictor involving the sum of smooth functions of covariates [50]. The GAMs were performed following a two-stage approach on two key response variables: CATCH (monthly number of bonefish caught by guides) and PTRIPS or the proportion of positive trips with a bonefish catch. PTRIPS was then the calculated as the monthly proportion of trips with a bonefish catch out of all fishing trips reported by guides that month (i.e., number of trips that kept or release bonefish / total number of trips). PTRIPS was a metric of catch success based on all trips (i.e., species presence-absence), including trips that targeted bonefish and trips that targeted other species than bonefish, that allowed for the identification of mechanisms that determine the occurrence of species independently of abundance dynamics [51]. The catch GAM (CATCH_gam_) was fitted using a log link function and negative binomial error distribution, while the proportion of positive trips GAM (PTRIPS_gam_) was fitted using a log link function and a binomial error distribution. GAMs were assessed for zero-inflation (i.e., positively skewed data) and overdispersion [51].

We fitted several continuous and categorical variables as potential explanatory factors in both GAM models. The continuous and categorical variables considered have been used in other standardization studies, and have been shown to be useful in controlling for CPUE variation associated with both effort and fishing dynamics [16,18,52,53]. To assess potential temporal thresholds (i.e., break-points and non-linear changes) in catch and occurrence, ‘Year’ (YR), ‘Month’, ‘Hours fished’ (HRSF) and ‘Number of fishermen’ (NFMEN) were included as continuous variables. HRSF and NFMEN were multiplied and considered as an intercept offset, which provides an advantage over using densities or rates (i.e., CPUE) as response variables, by limiting fitted variables and confidence intervals within positive values, and allowing for heterogeneity (i.e., different spread of fitted values) within a negative binomial distribution [54]. The categorical variables ‘Area’, and ‘Season’ were included to account for spatiotemporal patterns. ‘Area’ described fishing areas 1–3 of Florida Bay (Fig 1). The factor ‘Season’, fitted as a random variable, described seasonal rainfall patterns in South Florida: ‘wet’ = June-November, and ‘dry’ = December-May.

Following the approach of Winker et al. [16], continuous principal coordinates derived from a series of Principal Coordinates Analysis (PCOs) performed on the composition of the catch were also used as explanatory variables in GAMs (i.e., ‘Direct Principal Component’ or DPC procedure). The inclusion of PCOs from the catch composition matrix can help adjust for the effect of temporal variation in fishing tactics, which is a very common characteristic of multispecies fisheries [16,52], such as the recreational catch-and-release fishery in Florida Bay. This procedure is based on the assumption that information on the direction and extent of the targeted effort can be found in the species composition of the catch [52]. This procedure also allows for controlling for complex interactions among the response variables and the abundance and occurrence of sympatric or allopatric species, and for potential variation in fishing behavior when working with multispecies fisheries data [16,52]. PCOs were performed separately based on the species catch structure (i.e., species catch Bray-Curtis dissimilarity matrix) and the proportion of species (i.e., species occurrence Bray-Curtis dissimilarity matrix) associated with the bonefish catch to allow for different ecological and angler-behavior effects influencing the variability in fishing tactics. Only the first two coordinates of each PCO were included in the GAMs.

Forward and backward procedures were then used to add variables to the CATCH and PTRIPS full models, and to obtain the most parsimonious GAMs (S1–S4 Tables). First, explanatory variables and interaction terms were included if the percent of deviance explained by adding the factor exceeded 5% and the χ^2^ test was significant (p ≤ 0.05; [18]). Then, the resulting model from this first step was simplified further by dropping terms in a step-wise manner, as indicated by a drop in the Akaike information criterion (AIC) relative to the previous model using the delta-AIC of less than 2 units as a selection rule [55]. Once the set of the fixed explanatory variables and interaction terms was identified, the influence of the ‘Seasonal’ factor as a random variable was examined (using Generalized Additive Mixed Models). The interactions effects included in the models considered the influence of YR x Covariates interactions (i.e., YR x HRSF, YR x NFMEN, and YR x PCO). Season was included as a random variable since we were not interested in the variation as a function of specific seasonal events, but instead in overall seasonal heterogeneity [56] as a function of distinct patterns of temperature and precipitation that influence tourism and related fishing activities (i.e., tournaments, guided trips), as well as the distributional patterns of bonefish within Florida Bay. If a mixed model was selected as the appropriate model structure (i.e., including season as a random variable/effect), we followed Zuur et al. [54] to further simplify the mixed model with a backward selection procedure using AIC (S4 Table).

GAMs were applied in R [57] with the package ‘mgcv’ [58]. Cubic regression splines were used as the penalized smoothing basis (R code: bs = “cr”), and a tensor product interaction was used to assess the contribution of two-way interaction effects of different covariates (R code = ti). Based on diagnostic tests in the mgcv package (gam.check), we selected a maximum of 5 dimensions of the bases (R code: k = 5) to represent the smooth terms within the GAMs. In addition, to control for any overfitting of the smoothing terms estimated by the unbiased risk estimator (UBRE) criterion, a gamma value of 1.4 (γ = 1.4) was also included in the GAMs [58].

In addition to the CATCH and PTRIPS models, the temporal trends in catch species richness and the proportion of trips that caught bonefish when bonefish were targeted (i.e., targeted catching success) were also assessed with GAMs. For the first variable, we assessed the richness of the catch for all trips that included bonefish. For the second variable, we used data from 1990–2014 since data on species targeted were collected beginning in 1990. These additional analyses were used to complement and validate bonefish relative abundance trends derived from the catch data and to further reveal changes in bonefishing behavior and effort patterns. Last, a breakpoint analysis was performed using the annual average fitted values from the CATCH_gam_ and PTRIPS_gam_ to identify the presence of drastic changes in the temporal trends of the bonefish annual catch and the proportion of positive fishing trips (i.e., quantify structural changes in the time series—[59]). We used the breakpoint analysis of strucchange package in R, which uses maximum likelihood to identify structural changes in parametric models [59,60]. The breakpoint analysis employed in this study tests the hypothesis that regression coefficients remain constant against the alternative that at least one coefficient varies over time using a series of F statistics for all potential change points in an interval and rejecting the null hypothesis if any of those statistics get too large [60].

## Results

Post-standardization, the final CATCH and PTRIPS models shared a similar structure, but with some differences in the interaction terms (Table 1, S1 Table). Both models included YR (by Area), total HRSF, total NFMEN, and the first coordinate of the PCOs (PCO1.1 and PCO1.2) as covariates in model selection. The mixed model using season as a random variable (CATCH^1^ + random(Season) in Table 1 and S4 Table) improved the CATCH model by lowering the AIC from 4115.0 to 1655.9. Adding the random structure also improved the homogeneity of the residuals. We simplified further the CATCH mixed model, with a backward procedure that identifies the model with the lowest AIC, resulting in a final model that included only the covariates and no interaction terms (CATCH^2^ + random(Season) in Table 1 and S4 Table). The final PTRIPS model with the lowest AIC did not have a mixed effect structure, excluded Month as a covariate, and all interactions terms.

**Table 1:** Summary of mixed model results for a) the CATCH and b) proportion of positive trips (PTRIPS) GAMS.

**a) Mixed model formulations for bonefish total catch (CATCH)**		
**Model**	**AIC**	**Adjusted R**^**2**^
CATCH^1^	4115.0	0.91
CATCH^1^ + random(Season)	1655.9	0.85
**CATCH**^**2**^ **+ random(Season)**	**1626.3**	**0.85**
**b) Mixed model formulations for proportion of positive trip (PTRIPS)**		
**Model**	**AIC**	**Adjusted R**^**2**^
**PTRIPS**^**1**^	**274.3**	**0.96**
PTRIPS^1^ + random(Season)	4672.6	0.93

Final selected models are in bold. See footnote for details on the structure of the starting (S1 Table 1) and final selected models. Variables included: Year (Yr), Month, hours fished (HRSF), number of fisherman (NFMEN), first and second axis of Principal Coordinate Analysis based on species abundance (PCO1 and PCO2) and presence (PCO1.2 and PCO2.2) in the catch, fishing area (Area, see Fig 1), and Season as random variable. Fixed variables in the CATCH mixed model (CATCH + random(Season)) were further reduced (CATCH^2^).

**CATCH**^**1**^ **Model**: Catch = offset(Effort) + Yr_byArea_+ Month + HRSF + NFMEN + PCO1_byArea_ + PCO2_byArea_ + PCO1.2_byArea_ + Yr:HRSF + Yr:NFMEN + Yr:PCO1 + Yr:PCO1.2 + Yr:Month

**CATCH**^**2**^ **Model**: CATCH = offset(Effort) + Yr_byArea_+ Month + HRSF + NFMEN + PCO1_byArea_ + PCO1.2_byArea_

**PTRIPS**^**1**^ **Model:** PTRIPS = offset(Effort) + Yr_byArea_+ HRSF + NFMEN + PCO1_byArea_ + PCO1.2_byArea_

**Fig 2:**
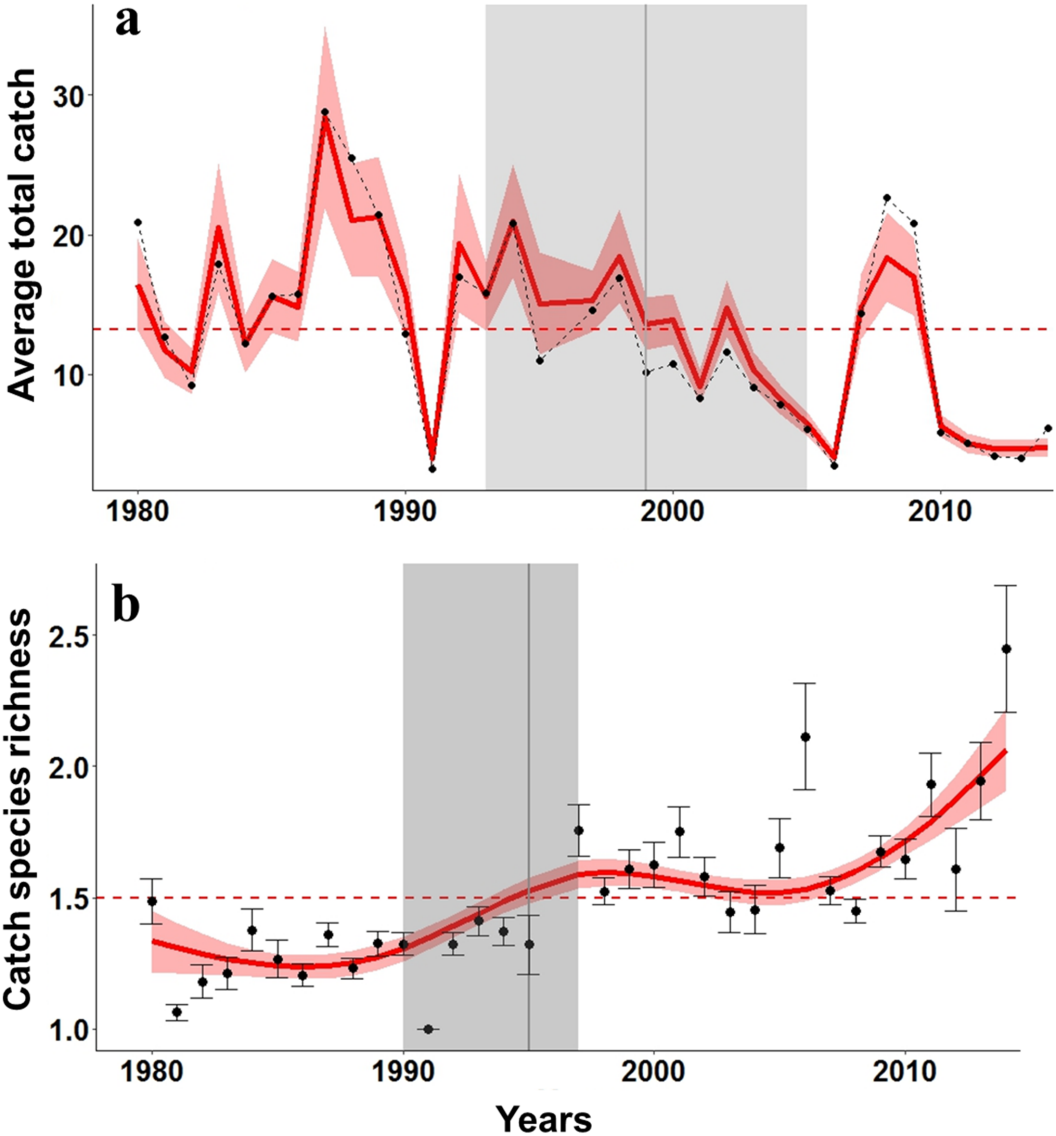
a) Fitted annual total catch of bonefish in Florida Bay in guided recreational trips, and b) fitted temporal trend in catch species richness for bonefish trips (number of caught along with bonefish) for 1980–2014 (yearly means and standard errors). Standardized values are shown in red, and upper and lower 95% confidence intervals are indicated by red shading. Breakpoints are indicated by grey vertical lines (see Table 2 for details) with 95% confidence intervals denoted by grey shading. Dotted horizontal lines illustrate mean values for the time series.

Using this final CATCH model, we found a declining pattern in bonefish catches reported by fishing guides in Florida Bay (Fig 2a). The pattern of decline, however, was not linear over the 34-year period examined. The breakpoint analysis identified 1999 as a major inflection point in the time series (Table 2). Bonefish catches were above average in the period 1980 to 1999, and below average post-2000. There was a 42% decrease in mean catch between 2000–2014, relative to 1980–1999 (F_1,32_ = 14.99, p < 0.001). Spanning the breakpoint, a steep monotonic decrease in catches is evident from 1995 to 2005 (Fig 2a). As bonefish catch declined, we observed an increase in the richness of the catch in bonefish trips (i.e., the number of species when bonefish was also caught, Fig 2b, Table 3). The breakpoint analysis determined 1995 as a point of major change in catch richness (Table 2). The period between 1980 and 1995 had a lower species richness relative to the richness reported after 1996 (34% increase, F_1,32_ p < 0.001).

**Table 2:** Breakpoint analysis results for annual bonefish catch, proportion of positive trips and catch richness associated with bonefish.

Response Variable	Estimated Breakpoint	Confidence Intervals (2.5–97.5%)		*F*	*p*
Catch	1999	1993	2005	15.88	<0.01
Proportion of positive trips	1989	1986	1990	31.76	<0.001
Catch richness	1995	1990	1997	36.64	<0.001

Breakpoints results are illustrated in figures as grey dotted line and shade area for 95% confidence intervals. The sup*F*-statistic (*F*) with estimated p-values (*p*) are presented for the null hypothesis of no structural change boundaries in *F* (see [59,60] for details).

**Table 3:** GAM results testing for temporal trends in the proportion of positive trips when bonefish was targeted and in catch species richness.

Y_*i*_	edf_year_	df_residuals_	*F*	*p*
Proportion of positive trips when bonefish was targeted	3.81	20.2	9.57	<0.001
Catch species richness	3.95	5042	47.05	<0.001

The results present the estimated and residuals degree of freedom (edf and df), *F*-statistics (*F*) and p-values (*p*).

**Fig 3:**
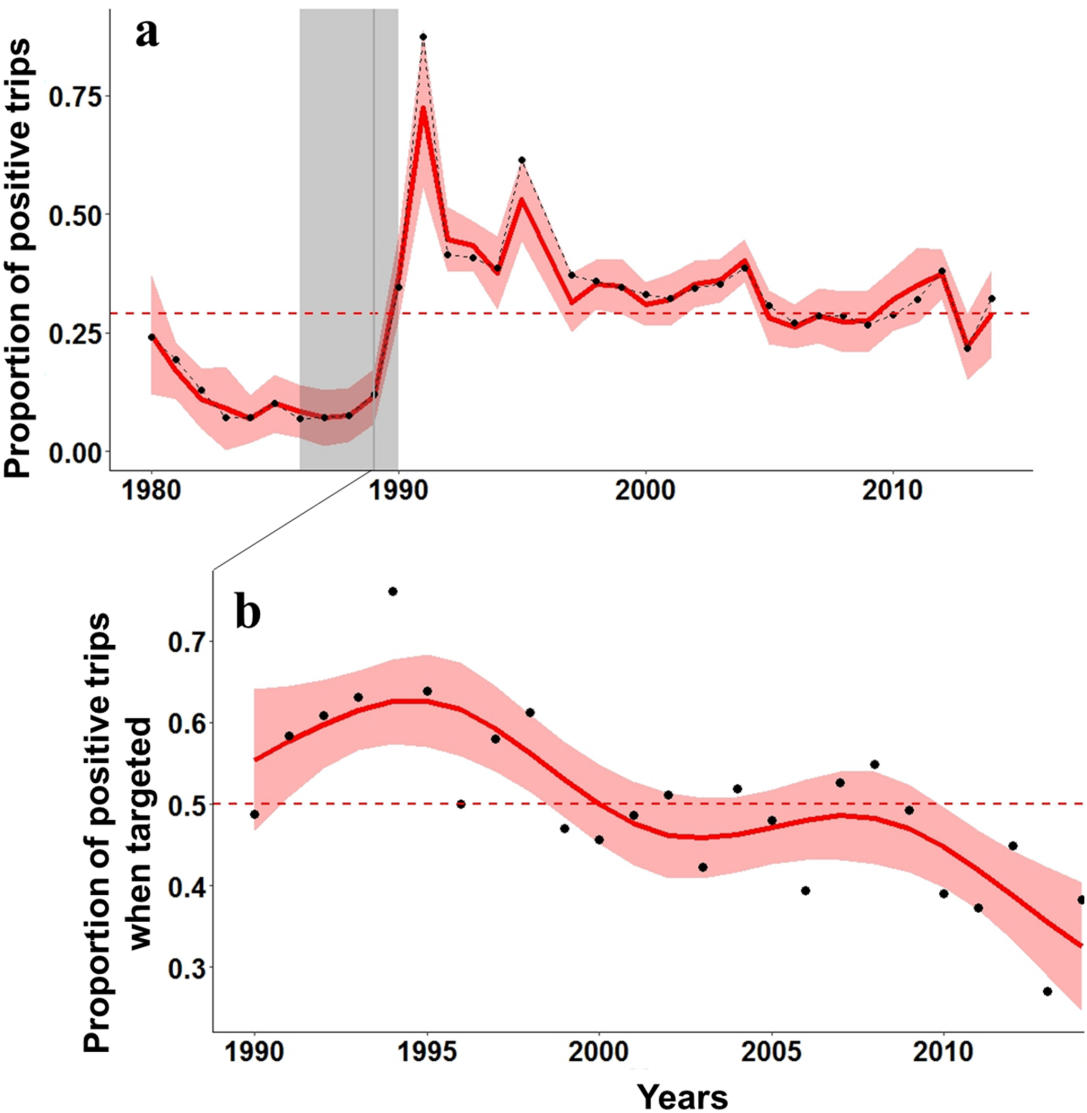
a) Bonefish fitted values for the proportion of positive trips (PTRIPS) from 1980–2014 and b) Fitted temporal trend in the proportion of positive trips when bonefish was the species targeted in a guided trip based on a GAM from 1990–2014. Fitted values are shown in red, with the upper and lower 95% confidence intervals indicated by red shading. A breakpoint in the PTRIPS time series is shown by a dotted grey line with 95% CI indicated by grey shading (see Table 2 for details). Horizontal dotted lines indicate means for each time series.

The trend obtained from the standardized PTRIPS (i.e., the proportion of positive trips with a bonefish catch) was similar to the bonefish catch trend (Fig 3a), with the exception of a period of low proportion of positive trips in the first part of the time series (1980 to 1989). A breakpoint was identified in 1989, after which the likelihood of catching a bonefish was higher (Table 2). However, this was followed by a monotonic decrease from 1991 onward (Fig 3a), with a 55% decrease in the mean occurrence between 1991 and 2014. Starting in 1990, guides began reporting whether bonefish was a targeted species on their fishing trips, allowing us to look at the success of catching a bonefish if targeted (i.e., the proportion of positive trips when bonefish was the targeted species). Here, three distinct periods of success at catching bonefish when targeted were identified (Table 3). From 1990 to 1998, on average 60% of the time guides successfully reported catching bonefish, followed by an intermediate period (1999–2009), where guides reported on average 48% catching success, and a lower period between 2011 and 2014 where success was only 37% (Fig 3b).

## Discussion

There is increasing evidence that, similar to commercial fisheries, recreational fisheries can be prone to severe declines and collapse [1,12,13]. However, data limitations make it a challenge to conduct stock assessments and quantify resilience in these recreational fisheries [61]. Using the data-limited bonefish catch-and-release fishery of Florida Bay, this study illustrates the utility of using different catch indices derived from FDD (e.g., catch, species richness associated to the catch, proportion of positive trips) to reconstruct historical abundance trends and determine, with a certain degree of confidence, major shifts in the trajectory of catch time-series. Analyses of bonefish catches in ENP guide reports pointed to a decline in bonefish catch and to changes in bonefishing effort in Florida Bay since the early 1980s. From these data, we identified three phases in the bonefish recreational fishery over the past 40 years: 1980–1988, 1989–1999, and 2000–2014. Bonefish catches in guided trips were increasing and highest in the first phase, decreasing with some stability in the second phase, and lowest in the third phase, with a shift to a declining trend between 1995 and 1999. Trends in the proportion of a bonefish positive trip and catching success when bonefish were targeted by guided trips followed the trends in catch, indicating declines and suggesting major population changes and/or shifts in bonefishing effort dynamics (i.e., fishing allocation time, spatial distribution, incorporation of alternative fisheries) starting in 1999–2000.

Despite their utility in quantifying and assessing changes in the abundance of fishery species, as illustrated by this study and others, it is worth nothing that FDD are subject to various biases and limitations. For example, the number of reports was limited in some years, especially at the beginning of the time-series, which could have produced anomalies in the CPUE and affected our assessment of temporal trends. In addition, FDD are subject to potential biases since they are inherently affected by fishing dynamics and angler behavior, which may cause CPUE to deviate from abundance, resulting at times in spurious inferences [15,62]. Following best practices in working with FDD, we standardized the catch data [15,16,47] by using a series GAMs that accounted for variation in temporal (e.g., month, season), spatial (e.g., fishing areas), catch structure associated with shifting tactics (e.g., PCO axes), and effort dynamics (e.g., hours fished, number of fishermen). These standardizations allow for extracting underlining patterns in FDD since they account for variation in fishing behavior and other known sources of variability [15]. The GAMs, however, did not include other possible variables that may have influenced fishing effort such as the socioeconomic and other environmental factors conditioning fishing trips (e.g., gas and market prices, regional economic indicators, storms etc.), also shown to be important, but more rarely accounted for [23,46].

Nevertheless, the concordance among the FDD indices of catch, species richness and the proportion of positive trips provided confidence in a pattern of a declining trend for bonefish in Florida Bay starting in the late 1990s. Importantly, these results agree with findings and angler concerns previously reported [2,33]. The only other FDD study of bonefish in the region, which used tournament records in the Florida Keys to develop an index of bonefish abundance, showed a declining trend between 1997 and 2010, which the authors attributed to increasing fishing pressure [23]. In a survey of fishing guides (n = 171), Larkin et al. [2] reported that half of the respondents surveyed in 2001 perceived a decline in the bonefish population in the Florida Keys. In a follow-up survey of the most experienced bonefish guides (n = 64), Frezza and Clem [33] reported a 78% decline in bonefish abundance in Florida Bay, higher than reported for any other area in South Florida, particularly for the period 2001–2012. Thus, our study contributes additional resolution to the dynamics of bonefish populations in South Florida, emphasizing a declining trend starting in the late 1990s.

We hypothesized that the pattern and timing of decline documented in our analyses could result from four key mechanisms: 1) shifts in fishing effort and angler behavior; 2) fishing pressure effects (i.e., indirect or direct effects of fishing mortality); 3) habitat/environmental effects; and 4) multiple interactions among these factors [4,12,63]. Previous work has indicated that major shifts in catch similar to the one observed in the bonefish FDD may be associated with changes in fishing behavior and effort dynamics [4,13]. For instance, sharp declines in catch can occur after giving-up density is reached; that is, the density or stock size that causes anglers to start abandoning the fishery resource [4]. Indeed, the significant increase in species richness associated with the bonefish catch after 1999 (Fig 2b) suggests that fishing and effort dynamics shifted as bonefish catches and positive trips declined. Similarly, Frezza and Clem [33] reported a 37% decrease in effort over time by guides interviewed in their survey. However, when looking at the records that reported targeting bonefish, the success of catch also declined after 2000, suggesting that the reduction in catch was mostly associated with changes in abundance.

There are numerous examples of how fishing pressure can directly influence the abundance and diversity of exploited species [64–66], including among recreational fisheries [1,12,67]. For bonefish, previous studies had reported relatively low fishing mortality for South Florida [2,23,68], although higher mortality has been observed in the Bahamas due to post-release predation by sharks and physiological effects (up to 40%; [69–71]). Nevertheless, based on two stock assessment models (i.e., a stochastic age-independent continuous population model and a catch-free assessment model), Larkin [23] estimated an increasing trend in fishing mortality since the mid-1980s in Florida Bay and the Keys, and suggested that even relatively low mortality could reduce bonefish stock abundance if effort and releases were high. The 1999 breakpoint observed in our FDD coincides with the point where annual mortality estimated by Larkin [23] surpassed 10%. Furthermore, for long-lived fish species such as bonefish (19–21 years, [31]), relatively low mortality may reduce stock abundance by compounding over time (i.e., accumulating and intensifying), resulting in non-linear declines [67].

It is a challenge, given the current data and models, to determine whether changes in bonefish populations may have occurred due to fishing mortality or recruitment effects or a combination of the two. Stocks can become susceptible to depletion through local recruitment overfishing (i.e., a fishing level that reduces recruitment of the exploitable stock) and/or lack of regional connectivity strength (i.e., depletion of a regional stock; [72,73]). In Florida Bay, the abundance of spawning-capable bonefish could have been reduced to such an extent that recruitment was reduced to levels that could not compensate for fishing effects. Bonefishing in Florida became officially catch-and-release only after 2013 (a one fish bag limit was allowed prior, mostly as trophy catch). A survey study showed that anglers perceived a major shift in bonefish size after 2000 (from 8-10lbs to 2-6lbs), indicating the possibility of an erosion of the cohort of larger bonefish that could result in recruitment overfishing [74]. The likelihood of local recruitment overfishing should be tested using simulations and population dynamic modeling (e.g., [75,76]). Alternatively, South Florida bonefish populations may be dependent on regional recruitment that may be eroded due to non-local fishing, harvest, or other large-scale habitat or environmental effects. In other areas in the Caribbean basin, bonefish are being harvested without any management oversight [27], thus potentially reducing the importance of these areas as sources of larvae to South Florida bonefish populations. For other taxa, such as lobsters, corals and reef fishes, Florida is typically considered a sink with a high degree of self-recruitment and high larval retention [77–79]. Whether the same is true for bonefish is unknown, but ongoing studies of genetic population structure should shed light on this issue (Adams et al., unpub. data).

Changes in seascape structure at broad spatial scales (e.g., the mosaic of seagrass habitat patches) can regulate differential responses in the demography of marine species [80–82], influencing the productivity of fisheries in coastal environments [83]. Starting in 1987, Florida Bay experienced a major drought-related seagrass die-off that affected 30% of the bay and triggered long-term alterations, including losses and changes in seagrass cover, algal blooms, sponge mortality, and reductions in spiny lobster landings and shrimp populations (i.e., one of the main bonefish prey items) [28,37,84]. These multiple interacting disturbances may have affected bonefish numbers and their recreational catches in a number of ways. For example, changes in angler behavior and effectiveness can be influenced by a number of factors such as: poor water clarity (i.e., bonefishing is largely a sight-based fishery); redistribution of bonefish to suboptimal areas and emigration; relocation of fishing effort (i.e., potentially leading to hyperstability); alterations to bioenergetics, trophic dynamics and predation risk (including post-release mortality); and reduced habitat quality for recruits. Both quantitative assessments (Larkin [23] and this study) pointed to a declining period in bonefish catch during and immediately after the seagrass die-off (i.e., a negative slope from 1988 to 1992), supporting the premise for an immediate role of this disturbance event, but further analyses relating bonefish catch with seagrass spatiotemporal dynamics and other associated environmental parameters are needed.

Finally, recent work points to the importance of the interactive effects of environmental factors and fishing on fisheries resilience [63,85]. Good examples of this include cases where truncation of the population structure due to fishing increases vulnerability to unfavorable environmental conditions by reducing the number of resistant age cohorts or those with a higher capacity to regenerate subsequent populations [4,63,85]. It is altogether plausible that post-release mortality may have accentuated immediately or years after the seagrass die-off disturbance (i.e., through changes in fish fitness due to habitat loss, fragmentation and associated effects on prey-predator dynamics) to exacerbate the bonefish decline. However, this remains unresolved. Lagged, interacting and accumulating effects could explain why the catch stabilized following the seagrass die-off until catch later shifted to below average conditions in the late 1990s (e.g., hyperstability, [4,62]). Linking the FDD and indices of abundance time series to drivers is the subject of ongoing work. Further, a similar hypersalinity and drought event in summer 2015 is currently unleashing another wave of seagrass die-off and algal blooms [30], providing an opportunity to more closely examine the effects of these major disturbances events on the structure and resilience of the Florida Bay recreational fisheries. In sum, our study shows that by assessing FDD with different metrics, we were able to reconstruct the pattern of catch and bonefishing effort, allowing for increased temporal resolution of the abundance dynamics of a data-limited fishery, and for the development of hypotheses about possible mechanisms causing population decline. The data-limited nature of most recreational fisheries, and the increase in a use of catch-and-release as a fisheries management strategy, highlight the need to develop further data integration approaches and tools that help assess fish population trends and the overall sustainability of recreational fisheries.

## Supporting information

S1 Table Process of initial full model construction. Variables included: Year (Yr), Month, hours fished (HRSF), number of fisherman (NFMEN), first and second axis of Principal Coordinate Analysis based on species abundance (PCO1 and PCO2) and presence (PCO1.2 and PCO2.2), fishing area (Area, see Fig 1), wet or dry season (Season) and weather bonefish was target or not (Target). NA resulted for models that did not converged. The final selected models where identified using a backward selection process (See S2 and S3 Tables for details) and illustrated below each table.
(DOCX)

S2 Table Model selection process to simplify the initial full CATCH model. Variables included: Year (Yr), Month, hours fished (HRSF), number of fisherman (NFMEN), first and second axis of Principal Coordinate Analysis based on species abundance (PCO1 and PCO2) and presence (PCO1.2 and PCO2.2). Asterisks are illustrating the variables that were included in each candidate model. We selected the model with the minimum AIC (i.e., equal to 0 delta AIC) as the final model for subsequent analyses (highlighted row).
(DOCX)

S3 Table Model selection process to simplify the initial full PTRIPS model. Variables included: Year (Yr), Month, hours fished (HRSF), number of fisherman (NFMEN), first and second axis of Principal Coordinate Analysis based on species abundance (PCO1 and PCO2) and presence (PCO1.2 and PCO2.2). Asterisks are illustrating the variables that were included in each candidate model. We selected the model with the minimum AIC (i.e., equal to 0 delta AIC) as the final model for subsequent analyses (highlighted row).
(DOCX)

S4 Table Process to simplify the initial CATCH mixed model (CATCH^1^ + random(Season)). This process was performed after identifying the mixed model as the most fitted model structure for CATCH. Variables included: Year (Yr), Month, hours fished (HRSF), number of fisherman (NFMEN), first and second axis of Principal Coordinate Analysis based on species abundance (PCO1 and PCO2) and presence (PCO1.2 and PCO2.2). Asterisks are illustrating the variables that were included in each candidate model. We selected the model with the minimum AIC (i.e., equal to 0 delta AIC) as the final model for assess the temporal trend in bonefish catch (highlighted row).
(DOCX)

S5 Table Index of catch and proportion of positive trips obtained from GAM models (i.e. fitted values: CATCH.mean.fit and PTRPS.mean.fit), and nominal catch and proportion of positive trips (i.e., raw means) from 1980 to 2014. The 95% confidence intervals (Upper- and Lower Bound) and standard errors (SE) of the fitted values are also listed for each model (CATCH and PTRIPS).
(DOCX)

S6 Table Mean species richness associated with bonefish catch from 1980 to 2014. Also listed the number of observations (N), the minimum and maximum species richness (Min and Max species richness) and the standard error of the mean values.
(DOCX)

S7 Table Proportion of positive trips when targeted from 1980 to 2014. The proportion consisted of the total number of trips that caught bonefish when targeted (Total of positive trips) divided by the total of trips that reported bonefish as the main targeted species (Total of targeted trips).
(DOCX)
