# Supplementary material for: Quantitative assessment of a data-limited recreational bonefish fishery using a time-series of fishing guides reports
Source: PLoS One. 2017 Sep 11;12(9):e0184776. doi: 10.1371/journal.pone.0184776 (PMC5593181; doi:10.1371/journal.pone.0184776)

**Supporting Information Table 1 (S1 Table).** Process of initial model construction. Variables included: Year (Yr), Month, hours fished (HRSF), number of fisherman (NFMEN), first and second axis of Principal Coordinate Analysis based on species abundance (PCO1 and PCO2) and presence (PCO1.2 and PCO2.2), fishing area (Area, see Figure 1), wet or dry season (Season) and weather bonefish was target or not (Target). NA resulted for models that did not converged. The final selected models where identified using a backward selection process (See S2 Table and S3 Table for details) and illustrated below each table.


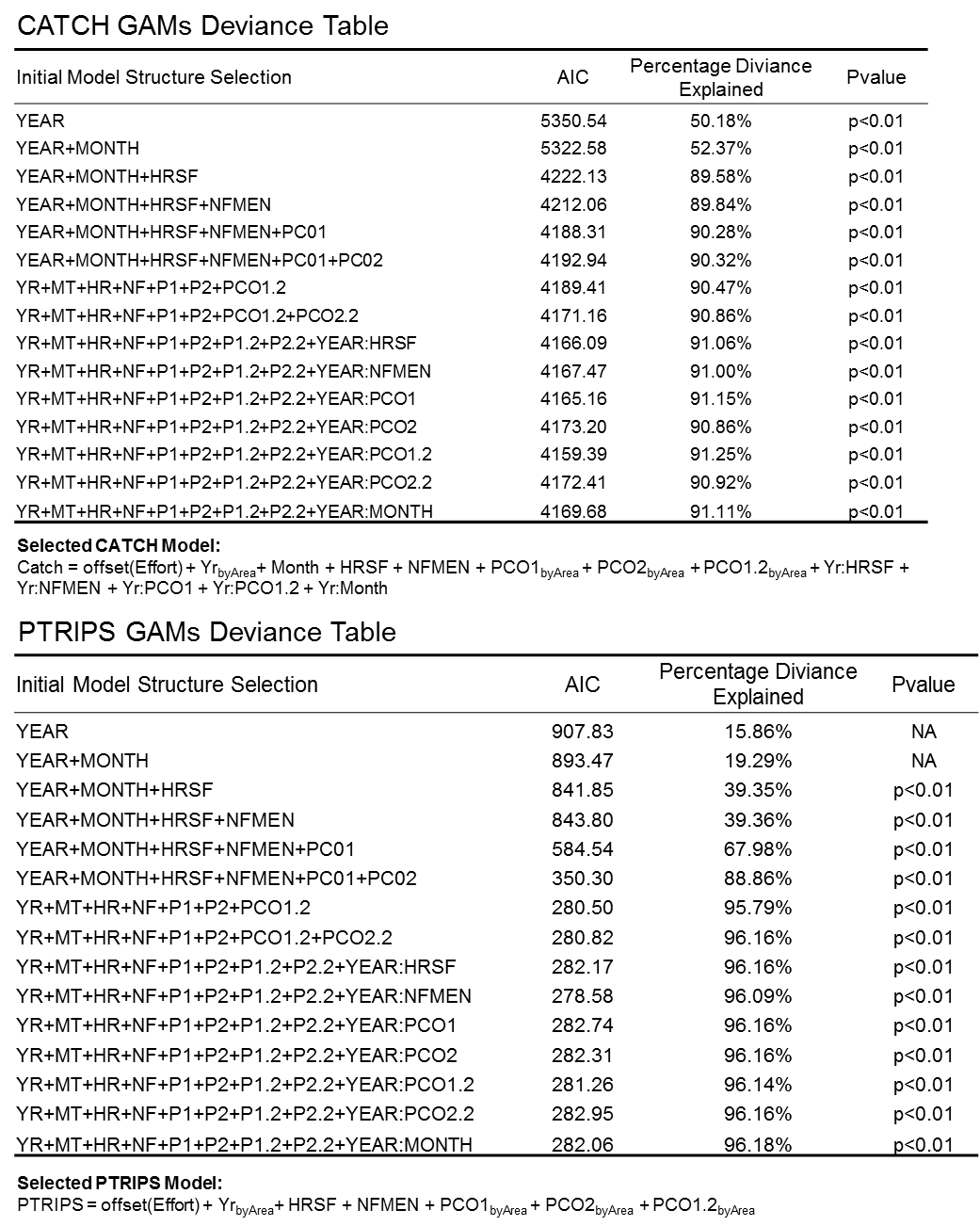

Supplement: S1 Table — Variables included: Year (Yr), Month, hours fished (HRSF), number of fisherman (NFMEN), first and second axis of Principal Coordinate Analysis based on species abundance (PCO1 and PCO2) and presence (PCO1.2 and PCO2.2), fishing area (Area, see Fig 1), wet or dry season (Season) and weather bonefish was target or not (Target). NA resulted for models that did not converged. The final selected models where identified using a backward selection process (See S2 and S3 Tables for details) and illustrated below each table. (DOCX) [file pone.0184776.s001.docx]
