# Supplementary material for: Quantitative assessment of a data-limited recreational bonefish fishery using a time-series of fishing guides reports
Source: PLoS One. 2017 Sep 11;12(9):e0184776. doi: 10.1371/journal.pone.0184776 (PMC5593181; doi:10.1371/journal.pone.0184776)
Supplement: S3 Table — Variables included: Year (Yr), Month, hours fished (HRSF), number of fisherman (NFMEN), first and second axis of Principal Coordinate Analysis based on species abundance (PCO1 and PCO2) and presence (PCO1.2 and PCO2.2). Asterisks are illustrating the variables that were included in each candidate model. We selected the model with the minimum AIC (i.e., equal to 0 delta AIC) as the final model for subsequent analyses (highlighted row). (DOCX) [file pone.0184776.s003.docx]

**S3 Table**. **Model selection process to simplify the initial full PTRIPS model.** Variables included: Year (Yr), Month, hours fished (HRSF), number of fisherman (NFMEN), first and second axis of Principal Coordinate Analysis based on species abundance (PCO1 and PCO2) and presence (PCO1.2 and PCO2.2). Asterisks are illustrating the variables that were included in each candidate model. We selected the model with the minimum AIC (i.e., equal to 0 delta AIC) as the final model for subsequent analyses (highlighted row).
