# Supplementary material for: Quantitative assessment of a data-limited recreational bonefish fishery using a time-series of fishing guides reports
Source: PLoS One. 2017 Sep 11;12(9):e0184776. doi: 10.1371/journal.pone.0184776 (PMC5593181; doi:10.1371/journal.pone.0184776)
Supplement: S5 Table — The 95% confidence intervals (Upper- and Lower Bound) and standard errors (SE) of the fitted values are also listed for each model (CATCH and PTRIPS). (DOCX) [file pone.0184776.s005.docx]

**S5 Table**. **Index of catch and proportion of positive trips obtained from GAM models (i.e. fitted values: CATCH.mean.fit and PTRPS.mean.fit), and nominal catch and proportion of positive trips (i.e., raw means) from 1980 to 2014.** The 95% confidence intervals (Upper- and Lower Bound) and standard errors (SE) of the fitted values are also listed for each model (CATCH and PTRIPS).
