# Supplementary material for: Quantitative assessment of a data-limited recreational bonefish fishery using a time-series of fishing guides reports
Source: PLoS One. 2017 Sep 11;12(9):e0184776. doi: 10.1371/journal.pone.0184776 (PMC5593181; doi:10.1371/journal.pone.0184776)
Supplement: S6 Table — Also listed the number of observations (N), the minimum and maximum species richness (Min and Max species richness) and the standard error of the mean values. (DOCX) [file pone.0184776.s006.docx]

**S6 Table**. **Mean** **species richness associated with bonefish catch from 1980 to 2014.** Also listed the number of observations (N), the minimum and maximum species richness (Min and Max species richness) and the standard error of the mean values.
