# Supplementary material for: Quantitative assessment of a data-limited recreational bonefish fishery using a time-series of fishing guides reports
Source: PLoS One. 2017 Sep 11;12(9):e0184776. doi: 10.1371/journal.pone.0184776 (PMC5593181; doi:10.1371/journal.pone.0184776)
Supplement: S7 Table — The proportion consisted of the total number of trips that caught bonefish when targeted (Total of positive trips) divided by the total of trips that reported bonefish as the main targeted species (Total of targeted trips). (DOCX) [file pone.0184776.s007.docx]

**S7 Table**. **Proportion of positive trips when targeted from 1980 to 2014.** The proportion consisted of the total number of trips that caught bonefish when targeted (Total of positive trips) divided by the total of trips that reported bonefish as the main targeted species (Total of targeted trips).
